# Effects of Directed Attention on Subsequent Processing of Emotions: Increased Attention to Unpleasant Pictures Occurs in the Late Positive Potential

**DOI:** 10.3389/fpsyg.2018.01127

**Published:** 2018-07-04

**Authors:** Yuming Chen, Dandan Zhang, Donghong Jiang

**Affiliations:** ^1^Department of Psychology, College of Psychology and Sociology, Shenzhen University, Shenzhen, China; ^2^Shenzhen Key Laboratory of Affective and Social Cognitive Science, Shenzhen University, Shenzhen, China; ^3^Center of Psychological Counseling, Shenzhen University, Shenzhen, China

**Keywords:** directed attention, attention history, emotion, early posterior negativity (EPN), parietal late positive potential (LPP)

## Abstract

Directed attention is a fundamental mental resource for voluntarily managing the focus and direction of cognitive resources. The present study investigated how processing of unpleasant and neutral images is affected by emotion and previous directed attention. The results showed that there was enhanced early posterior negativity, anterior N2, and parietal late positive potential (LPP) in response to unpleasant pictures compared to neutral pictures. Furthermore, attention history (i.e., whether stimuli were previously attended to) modulated the amplitudes of the anterior N2 and parietal LPP. Most notably, an interaction between attention history and emotion was found in the LPP: pictures with an ‘attended history’ evoked larger LPP amplitudes than pictures with an ‘unattended history,’ but this effect was only significant for unpleasant pictures (not for neutral pictures). These results suggest that directed attention to affective pictures facilitates subsequent neural processing of these pictures, and that this effect was amplified by unpleasant emotions experienced in the LPP. The current findings provide further empirical evidence of a two-stage model of emotion-attention interaction.

## Introduction

Emotion is an important part of the human experience, where processing emotional stimuli is necessary for health and survival (Lang and Bradley, 2010). Directed attention (also called voluntary attention) is a fundamental mental resource for voluntarily managing the focus and direction of thoughts, and also regulates emotions (Morecraft et al., 1993; Dunning and Hajcak, 2009; Hajcak et al., 2009; Shaw et al., 2014; Dixon et al., 2017). Directed attention often occurs in a top-down or goal-directed fashion (Ferrari et al., 2008) and is associated with the prefrontal cortex (Schafer and Moore, 2011). Most previous studies that focused on the effect of directed attention on emotional processing examined the “instant” effect, i.e., the processing of emotional stimuli that appeared for the first time in a directed attention task (referred to as “the directed attention phase” in this study). Very few studies have addressed the issue of emotional processing in the context of a history of directed attention, i.e., the effect of attention on emotional processing when emotional stimuli appeared for the second time after the directed attention phase (referred to as “the re-exposure phase” in this study). Two factors influence visual processing of previously presented stimuli in the re-exposure phase, namely directed attention history and emotion-driven attention. The latter factor reflects the phenomenon wherein emotional, and particularly unpleasant, stimuli usually capture more attention than neutral stimuli because they are inherently significant (Bradley et al., 2003; Olofsson et al., 2008). These considerations raise two important research questions. First, is the effect of directed attention on emotional processing similar between the re-exposure and directed attention phases? Second, is the effect different between unpleasant and neutral stimuli?

Many previous studies using event-related potentials (ERPs) have examined the relationship between directed attention and emotional processing during the directed attention phase. Early posterior negativity (EPN) and late positive potential (LPP) are thought to be two reliable markers of emotional processing (De Cesarei et al., 2017). The EPN is a negative-going wave in occipito-temporal sites that usually peaks at 200–300 ms post-stimulus onset (Schindler et al., 2015). It has been demonstrated that, within the EPN time window, directed attention affects the processing of emotional stimuli, but no interaction has been found between directed attention and emotion (Schupp et al., 2007; Kissler et al., 2009; Schindler and Kissler, 2016). The LPP is a positive-going slow-wave that is maximal at central-parietal sites and often occurs from 300 ms after stimulus presentation (Thiruchselvam et al., 2011). Directed attention affects the processing of unpleasant pictures in the LPP time window. For example, Hajcak et al. (2009, 2013) instructed participants to focus on either high- or low-arousing areas of unpleasant pictures, indicated by a circle (Dunning and Hajcak, 2009; Hajcak et al., 2013) or a low/high tone (Hajcak et al., 2009), and found that LPP amplitudes decreased when participants focused on the low-arousing areas (note: the term *arousal* refers to the physiological and psychological state of being awoken). Furthermore, an interaction between directed attention and emotion has been reported in LPP. For example, Schupp et al. (2007) manipulated directed attention to certain types of stimuli by having participants count pleasant, unpleasant, or neutral pictures and found larger LPP amplitudes for targets relative to non-targets, where the effect was larger for emotional stimuli than for neutral stimuli, indicating that directed attention effect was augmented for emotional stimuli. Schindler and Kissler (2016) employed emotional words in a similar task and found that the difference between the LPP evoked by emotional compared to neutral words was substantially larger when the words were targets versus non-targets, indicating that the effect of emotion was augmented for targets.

More importantly, two ERP studies have investigated the effects of directed attention history on emotional processing in the re-exposure phase (Thiruchselvam et al., 2011; Paul et al., 2016) and showed that the LPP amplitude increased in response to unpleasant pictures with a distraction instruction (i.e., to generate neural thoughts unrelated to the image) history compared to those with an attention instruction history when participants are re-exposed to these images as targets (i.e., participants were instructed to attend to the images and rate the unpleasantness of them in the re-exposure phase). Here, enhancement of LPP amplitudes was due to the distraction history hindering the emotional processing of the pictures. Thus, these pictures required more cognitive resources when they were presented as targets during the re-exposure phase. Notably, previously experienced stimuli may appear again, not only as targets but also as distractors, in everyday life. The findings reported by Paul et al. (2016) and Thiruchselvam et al. (2011) may not apply to the effect of directed attention history on the emotional images when participants are re-exposed to these images as distractors, as processing of the target differs from that of the distractor (Debener et al., 2005).

In the present study, we examined how directed attention history affects the processing of unpleasant and neural images re-presented but presented as distractors. We used a study–test paradigm combined with a cued/uncued task during the directed attention phase, and an oddball task during the re-exposure phase. In the cued/uncued task, we directed the attention of participants to a neutral or unpleasant image while the two were presented simultaneously. Subsequently, all images were sequentially presented as infrequent distractors in a three-stimulus oddball task. We focused on three ERP components elicited by the neutral and unpleasant distractor images in the re-exposure phase. The first one was the EPN, which was shown to reflect the effects of directed attention and emotion independently during the directed attention phase (Schupp et al., 2007; Kissler et al., 2009; Schindler and Kissler, 2016). Accordingly, we hypothesized that similar independent effects would occur during the re-exposure phase. The second component was the parietal LPP. It was expected that images with an ‘unattended history’ would elicit smaller LPP amplitudes than those with an ‘attended history’, where in the former case, participants should be able to ignore such images, and thus show reduce the emotional processing, when they are subsequently presented as distractors. Furthermore, previous studies have shown an interactive effect between directed attention and emotion on LPP during the directed attention phase (Schupp et al., 2007; Schindler and Kissler, 2016). In line with this result, we expected that the interactive effect would still occur during the re-exposure phase, with greater directed attention history effect for unpleasant images compared to neutral images. In addition to the EPN and LPP, this study also examined the anterior N2 component. Previous studies have found that N2 is associated not only with detecting novelty (Ferrari et al., 2010; Zheng et al., 2010), but also with detecting emotional significance (Jiang et al., 2013; Yuan et al., 2015). We expected that the images with an ‘unattended history’ would elicit a larger N2 because they would be perceived as more novel than those with an ‘attended history’. It was also expected that unpleasant images would elicit a larger N2 amplitude than neutral images.

## Materials and Methods

### Participants

Thirty healthy students were recruited from Shenzhen University and paid for their participation. All were right-handed and had normal or corrected-to-normal vision, as well as normal color vision (examined with Ishihara color tables; Ishihara, 1972). Participants had no history of neurological or psychiatric disorders. Four subjects were excluded from analyses due to low accuracy rate (< 50%) while two subjects were excluded due to exceedingly high artifacts in the electroencephalography (EEG). The final sample consisted of 24 subjects (12 males; age range = 18–24 years). All participants provided written informed consent prior to the experiment. The experimental protocol was approved by the Ethics Committee of Shenzhen University and was in compliance with the 1964 Helsinki Declaration and its later amendments, and this study was performed in strict accordance with the approved guidelines.

### Stimuli

The fixation point was a 0.3° × 0.3° cross at the center of the computer monitor. Because of a cultural bias in the International Affective Picture System with respect to Chinese participants (Huang and Luo, 2004), the emotional pictures (3.0° × 2.2° visual angle) were selected from the Chinese Affective Picture System (CAPS) (Bai et al., 2005) and included 80 unpleasant (e.g., scene of a car accident) and 80 neutral (e.g., a city landscape) pictures. Each picture had been assessed in a previous survey by a large sample of Chinese participants for its valence (i.e., ranging from unpleasant to pleasant), arousal (i.e., ranging from calm to excited) and dominance (ranging from in-control to controlled) quality using a nine-point scale (Bai et al., 2005). The emotional valence of unpleasant pictures was significantly lower than that of neutral pictures [2.58 ± 0.66 vs. 5.10 ± 0.21, *F*(1,158) = 1062, *p* < 0.01], and unpleasant pictures were associated with significantly greater emotional arousal than were neutral pictures [6.01 ± 0.61 vs. 4.04 ± 0.78, *F*(1,158) = 315.8, *p* < 0.01]. Additionally, the dominance of unpleasant pictures was significantly lower than that of neutral pictures [2.65 ± 0.47 vs. 5.97 ± 0.83, *F*(1,158) = 964.6, *p* < 0.01]. All pictures were identical in size and resolution (100 pixels per inch), and the luminance, contrast, and spatial frequency were also counterbalanced between unpleasant and neutral conditions. A white star was used as a standard (2.0° × 2.0° visual angle) or target stimulus (2.2° × 2.2° visual angle) in the oddball task.

### Procedure

**FIGURE 1:**
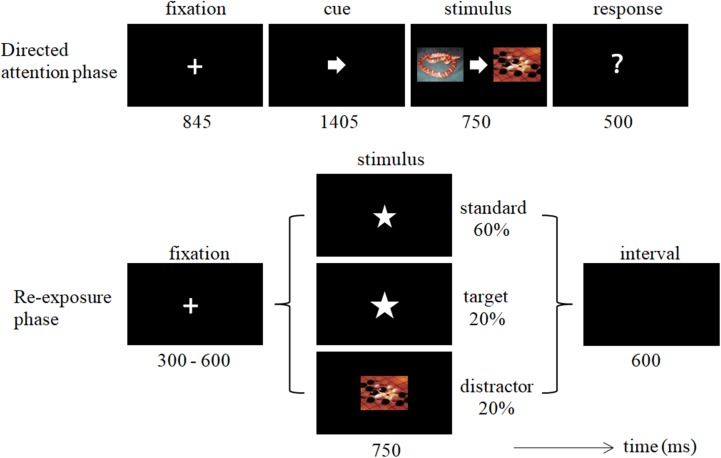
Illustration of experimental trials during the directed attention phase and re-exposure phase.

Participants were seated comfortably in a dim, sound-attenuated, and electrically shielded room facing a computer screen placed 100 cm in front of them. The experimental procedure consisted of two phases, i.e., the directed attention phase and the re-exposure phase (**Figure 1**). Before the formal experiment, each subject was given 10 practice trials for each phase to ensure understanding of the experimental task. The entire experiment took about 35 min.

In the directed attention phase, subjects were instructed to complete a cued/uncued task consisting of 80 trials. As described previously (Yamagata et al., 2000), each trial began with an 845-ms fixation, followed by a 1,405-ms arrow cue. Then, one neutral and one unpleasant picture were presented to the left or right side of the cue for 750 ms. Participants were told that each of the two pictures beside the cue could have two emotional valence categories, unpleasant or neutral. They were required to attend to, and judge, as quickly as possible, the valence of the picture (neutral or unpleasant) indicated by the cue, by pressing the “F” or “J” key on the computer keyboard with the left or right index finger. The assignment of keys to unpleasant and neutral categories was counterbalanced across participants. All 80 unpleasant and 80 neutral pictures were used in the directed attention phase, and these were equally divided (the difference of valence, arousal and dominance were not significant, *ps* > 0.4) between attended (indicated by the cue) and unattended (not indicated by the cue) pictures at this phase. The attended and unattended pictures were counterbalanced between the left and right sides across trials and they were counterbalanced between the unpleasant and neural categories across subjects.

After a 5-min rest period, the participants performed an oddball task with three types of stimuli (60% standard, 20% target, and 20% distractor stimuli) in the re-exposure phase. The standard stimulus and the target stimulus were the same star, except that the target was 10% larger than the standard stimulus. The distractor stimuli were the 160 unpleasant/neutral pictures used in the directed attention phase. As shown in **Figure 1**, after a 300–600-ms fixation, a stimulus was presented for 750 ms, followed by an interval of 600 ms. Participants were instructed to respond to the target as quickly as possible by pressing a button with the right or left index finger (right and left fingers were counterbalanced across subjects) and to ignore the other stimulus. The subjects did not know in advance that the unpleasant/neutral pictures in the directed attention phase would reappear in this task. The re-exposure phase consisted of 800 trials, including 480 standard, 160 target, and 160 distractor trials.

### EEG Recording and Analysis

Electroencephalography and ocular movements were recorded with reference to the left mastoid and re-referenced offline relative to the average signals at the left and right mastoids by way of a 64-channel amplifier with a sampling frequency of 500 Hz (Brain Products, Gilching, Germany). EEG data were collected with electrode impedances kept below 5 kΩ. We recorded from 28 standard electrode sites: Fz, FCz, Cz, CPz, Pz, Oz, Fp1/2, F3/4, F7/8, FC3/4, FT7/8, C3/4, T7/8, CP3/4, P3/4, P7/8, and O1/2.

Ocular artifacts were removed from EEGs using the independent component analysis procedure implemented in Brain-Vision Analyzer 2.0 (Brain Products). The recorded EEG data were filtered with a 0.01–30 Hz finite impulse response filter with zero phase distortion. Filtered data were segmented (900-ms periods) by the type of distractor stimulus (neutral or unpleasant pictures previously attended to or not) at the re-exposure phase, beginning 150 ms prior to the onset of the distractor stimulus. ERP epochs with a “false alarm” response were excluded from analyses. All epochs were baseline-corrected with respect to the mean voltage during the 150 ms preceding stimulus onset, followed by averaging in association with the types of distractor stimulus. Trials in which the EEG exceeded a ± 70 μV range were rejected. The rejected trials were 1.00 ± 1.64, 1.17 ± 1.86, 1.21 ± 1.53, and 1.33 ± 1.61 for attended unpleasant, attended neutral, unattended unpleasant and unattended neutral conditions respectively. The rejected trials did not differ across conditions. No interpolated electrodes were used in this study.

This study focused the emotion-related ERP components (i.e., occipital EPN, anterior N2, and parietal LPP) elicited by distractor images during the re-exposure phase. ERP amplitudes were measured using different sets of electrodes in accordance with grand mean ERP topographies (see in Supplementary material) and established protocols. Mean EPN amplitude was calculated at occipital sites O1, O2, and Oz (Wheaton et al., 2013; Suess and Abdel, 2015) within a time window of 120–200 ms. Mean anterior N2 amplitude was calculated at electrode sites Fz, F3, F4, FCz, FC3, and FC4 (Folstein and Van Petten, 2008; Schomaker and Meeter, 2014) within 200–270 ms after stimulus onset. Mean parietal LPP amplitude was calculated at electrode sites Pz, P3, P4, CPz, CP3, and CP4 (Hajcak et al., 2009, 2013) between 550–750 ms after stimulus onset.

### Statistics

Statistical analyses were performed using SPSS 18.0. Two-way repeated-measures Analysis of variance (ANOVA) was performed on ERP measurements (EPN, N2, and LPP mean amplitudes), with attention history (previously attended to or not) and emotional valence (unpleasant or neutral) as the within-subject factors. Descriptive data are presented as means ± standard deviations. Significant interactions were analyzed using a simple-effects model. The significance level was set at 0.05. The interactions were assessed according to the Newman–Keuls *post hoc* means comparison procedure. Partial eta squared (η^2^) was calculated to determine the effect size of the ANOVA tests.

## Results

### Behavioral Data

Performance during the directed attention phase was assessed in terms of the rates of correct button presses. The total accuracy rate was 81.6 ± 6.9%, indicating that participants performed in accordance with the requirements of the cued/uncued task. The relatively low accuracy rate may due to the short response window (500 ms; **Figure 1**) in this study.

Oddball task performance was assessed in terms of hit rates and hit RTs. The hit rate was the proportion of correct button presses in target trials. The mean hit rate was 77.4% and the false alarm rate was 8.8%, indicating that participants did act in accordance with the instruction to respond to targets. The mean hit RT was 509.8 ± 48.2 ms.

Behavioral data of each subject is provided in the supplymentary material.

### EPN

**FIGURE 2:**
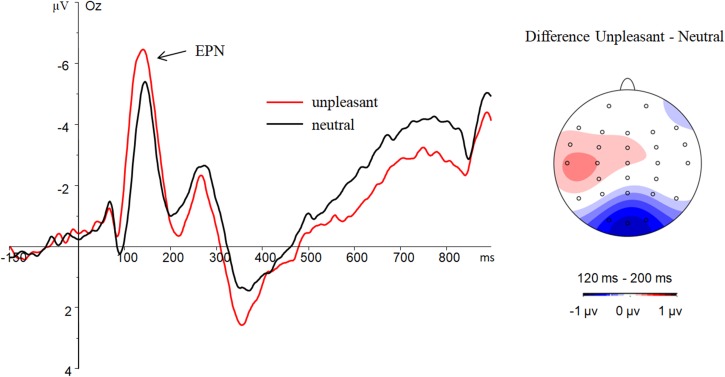
Results for main effects of emotion in the EPN. Grand average ERP waveforms at electrode Oz are displayed on the left. Difference topography of unpleasant minus neutral is displayed on the right.

The ANOVA showed a significant main effect of emotional valence on EPN amplitude [*F*(1,23) = 6.21, *p* = 0.02, η^2^= 0.21; **Figure 2**]; the EPN amplitudes for unpleasant images (-3.76 ± 3.94 μV) were larger than those for neutral images (-3.08 ± 4.01 μV). Neither the main effect of attention history [(*F*(1,23) = 0.13, *p* = 0.72, η^2^= 0.01] nor the interaction of emotional valence by attention history [(*F*(1,23) = 0.02, *p* = 0.89, η^2^ = 0.00] was significant.

### Anterior N2

**FIGURE 3:**
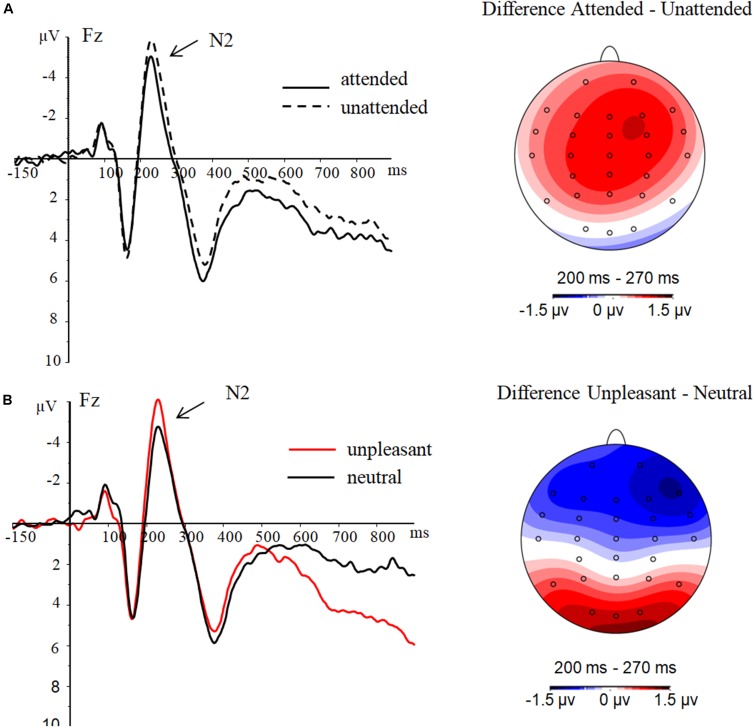
Results for the anterior N2. **(A)** Main effects of attention history. **(B)** Main effects of emotion. ERP waveforms are obtained at electrode Fz.

As shown in **Figure 3**, there was a significant main effect of attention history on mean anterior N2 amplitude [(*F*(1,23) = 4.81, *p* = .04, η^2^= 0.17]. The N2 amplitude under the attended history condition (-2.98 ± 0.93 μV) was smaller than that under the unattended history condition (-3.84 ± 1.05 μV). There was also a significant main effect of emotional valence on anterior N2 amplitude [(*F*(1,23) = 5.33, *p* = 0.03, η^2^= 0.19]. The N2 amplitude produced by unpleasant images (-3.83 ± 1.03 μV) was larger than that produced by neutral images (-2.99 ± 0.94 μV). There was no significant interaction between attention history and emotional valence [(*F*(1,23) = 1.05, *p* = 0.32, η^2^ = 0.04].

### Parietal LPP

**FIGURE 4:**
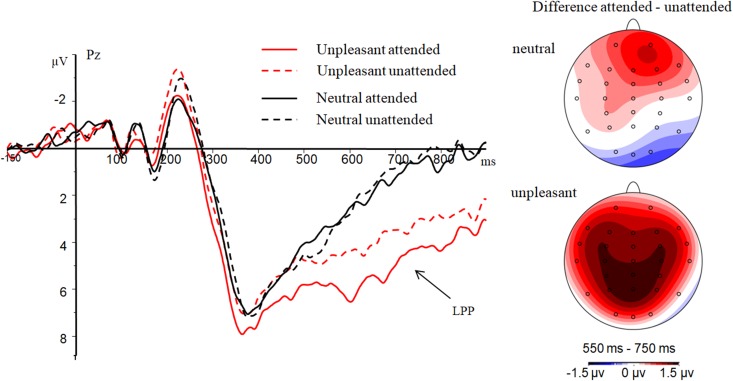
Results for the interaction effect in the LPP. Grand average ERP waveforms at electrode Pz are displayed on the left. Difference topographies of attended minus unattended condition are displayed on the right.

Emotional valence had a significant main effect on mean parietal LPP amplitude [*F*(1,23) = 35.46, *p* < 0.01, η^2^ = 0.61], with a more positive amplitude observed for unpleasant (5.86 ± 4.26 μV) than for neutral images (3.06 ± 3.63 μV). The mean parietal LPP amplitude was significantly affected by attention history [*F*(1,23) = 5.40, *p* = 0.03, η^2^= 0.19], with a greater amplitude for images with an attended history (4.91 ± 3.73 μV) compared with those with an unattended history (4.02 ± 4.06 μV). The interaction between attention history and emotional valence was significant [*F*(1,23) = 8.90, *p* = 0.01, η^2^= 0.28]. Further analysis showed that the effect of attention history was significant for unpleasant [*F*(1,23) = 8.46, *p* = 0.01, η^2^ = 0.27] but not for neutral [*F*(1,23) = 0.12, *p* = 0.74, η^2^ = 0.01] images (**Figure 4**). A larger LPP amplitude was observed for unpleasant images with an attended history (6.65 ± 4.38 μV) than for those with an unattended history (5.08 ± 4.53 μV).

## Discussion

This study assessed the effect of directed attention on the processing of emotional images in the re-exposure phase, and compared the effect between unpleasant and neutral stimuli. As expected, ERP measurements revealed an emotion-related EPN, anterior N2, and LPP. The effect of directed attention history was seen in the anterior N2 and parietal LPP. Most importantly, an interaction between attention history and emotion was reflected in the parietal LPP amplitude.

The result that larger amplitudes of the EPN and LPP (Schupp et al., 2003a, 2007; Flaisch et al., 2011; Horan et al., 2012; Bayer and Schacht, 2014), as well as of the anterior N2 (Jiang et al., 2013), were evoked by unpleasant images compared to neutral images is in line with previous studies. In addition, the larger LPP amplitudes for unpleasant compared to neutral images are consistent with previous repetition studies which found larger LPP amplitudes for unpleasant pictures than neutral ones when these pictures were repeatedly presented (Codispoti et al., 2007, 2016). The increase in these three ERP components reflects augmented selective perceptual encoding guided by unpleasant stimuli with a significant motivational impact (Schupp et al., 2003b; Lang and Bradley, 2010; Jiang et al., 2013). In addition, it was found that high-arousal stimuli evoked larger amplitudes of the N2, EPN and LPP than low-arousal stimuli (Rozenkrants and Polich, 2008). Therefore, unpleasant images take priority with respect to attentional resources because they are more arousing than neutral images.

For the anterior N2 and parietal LPP, attention history affected the processing of emotional pictures during the re-exposure phase. The N2 amplitude was associated with attention history and the effect was independent of emotion. In addition to the association with attention control (Dennis and Chen, 2007; Folstein and Van Petten, 2008), enhanced N2 was also observed in situations where attention was directed to novel stimuli (Folstein and Van Petten, 2008; Schomaker and Meeter, 2014). In line with the notion of detecting novelty, the observed larger N2 amplitudes for images with an unattended versus attended history may be due to the images not attended to during the directed attention phase being perceived as more novel during the re-exposure phase. This study did not find an effect of attention history on the EPN, which is inconsistent with previous studies (Schupp et al., 2007; Schindler and Kissler, 2016). There may be two reasons for this. First, previous studies focused on the effect of directed attention in the directed attention phase (Schupp et al., 2007; Schindler and Kissler, 2016), whereas this study investigated the effect in the re-exposure phase. Furthermore, in this study, the stimuli of interest were attended to, or not, indicated by a cue, and the processing of unattended and attended stimuli may be different from the processing of target and non-target stimuli during the directed attention phase, as in Schupp et al. (2007) and Schindler and Kissler (2016). Second, this study used a linked mastoid reference, which may have decreased the EPN effect (Junghofer et al., 2006); both Schupp et al. (2007) and Schindler and Kissler (2016) used an average reference.

The most important finding in this study was the interactive effect between attention history and emotion on the parietal LPP amplitude; that is, enhanced LPP amplitudes were observed for unpleasant pictures with an ‘attended history’ compared to those with an ‘unattended history,’ but the effect was not significant for neutral pictures. This result is in line with a previous study showing that directing attention to areas stimulating a high level of emotional arousal could enhance emotional processing at the directed attention phase (Dunning and Hajcak, 2009; Hajcak et al., 2009, 2013). The current findings indicate that the facilitated processing of unpleasant images associated with directed attention is also significant in the re-exposure phase. In addition, the observed interactive effect between attention history and emotion on the parietal LPP is in line with Schupp et al. (2007), who found that the attention effect was larger for emotional than neutral images during the directed attention phase. Furthermore, studies have suggested that arousal, not valence, affected the interaction of attention and emotion (Schindler and Kissler, 2016). One possible explanation for the interaction is that prior attention promoted emotional processing of unpleasant stimuli more so than neutral stimuli since unpleasant images were more arousing than neutral images, which then enhanced recognition memory during the re-exposure phase (Srinivasan and Gupta, 2010).

The current finding is not consistent with previous results showing that previous distraction enhances LPP for unpleasant images during the re-exposure phase (Thiruchselvam et al., 2011; Paul et al., 2016). The discrepancy may be due to different tasks between these studies. It has been demonstrated that task did affect the LPP amplitudes. For example, Ferrari et al. (2013) found that repetition enhanced the old–new LPP effect for both emotional and neutral pictures when there was an explicit recognition test during memory retrieval; however, the old–new LPP effect was only apparent for emotional pictures when there was a passively viewing task during memory retrieval. In this study, we investigated the effect of attention history on distractor images. Therefore, an ‘unattended history’ allowed the unpleasant images to be ignored when they were presented as distractors during the re-exposure phase. On the contrary, Paul et al. (2016) and Thiruchselvam et al. (2011) investigated the effect of attention history on target images during the re-exposure phase. In their studies, distraction history hindered the processing of emotional pictures so that more attentional resources were needed for these pictures when they re-appeared as targets. Second, the directed attention instructions were different between studies: the subjects were asked to use a distraction or attention strategy in Paul et al. (2016) and Thiruchselvam et al. (2011), whereas attention was guided by a cue in the present study. Third, the present study used a shorter interval between the directed attention and re-exposure phases [compared to 10 min in Paul et al. (2016) and 30 min in Thiruchselvam et al. (2011)]. It is likely that the effect of directed attention would vary by length of interval. Further research is needed to explore the effects of the interval between the two phases.

In summary, this study extends the two-stage model of emotion-attention interaction (Schupp et al., 2006, 2007), from the directed attention phase to the re-exposure phase. The first stage involves perceptual processing of emotionally significant information (reflected by the EPN and N2) and novel stimuli (reflected by the N2). The two perceptual processes are independently modulated during the re-exposure phase. The second stage involves elaborate and sustained attentional processing, where emotional significance and attention history synergistically modulate the processing of re-exposed stimuli (reflected by the LPP). The current findings have implications for the utility of directed attention in cognitive interventions: an unattended strategy in the directed attention phase is effective for weakening the emotional processing of unpleasant stimuli during the re-exposure phase when the unpleasant information is no longer useful (i.e., unpleasant stimuli occur as distractors after the directed attention phase).

## Author Contributions

YC and DJ managed the literature research, the study design, experimental studies, and data collection. DZ and DJ managed data analysis and interpretation, and wrote the initial draft of the manuscript. DJ created all figures. YC and DZ contributed equally to this work. All authors reviewed the manuscript. Correspondence and requests for materials should be addressed to DJ (email: jiangdh@szu.edu.cn).

## Conflict of Interest Statement

The authors declare that the research was conducted in the absence of any commercial or financial relationships that could be construed as a potential conflict of interest.
