# Supplementary material for: Effects of Directed Attention on Subsequent Processing of Emotions: Increased Attention to Unpleasant Pictures Occurs in the Late Positive Potential
Source: Front Psychol. 2018 Jul 4;9:1127. doi: 10.3389/fpsyg.2018.01127 (PMC6040230; doi:10.3389/fpsyg.2018.01127)
Supplement: Supplementary file 1 [file Data_Sheet_1.DOCX]

Supplementary Table. Behavioral data for all the 30 participants.

| Subject  number | Accuracy in habituation | Hit rate  in re-exposure | False alarm rate  in re-exposure | Include in analysis |
| --- | --- | --- | --- | --- |
| 3 | 0.8 | 0.7 | 0.02 | yes |
| 4 | 0.7 | 0.64 | 0.03 | yes |
| 5 | 0.925 | 0.79 | 0.24 | yes |
| 6 | 0.925 | 0.65 | 0.05 | yes |
| 7 | 0.8625 | 0.8 | 0.16 | yes |
| 8 | 0.7625 | 0.69 | 0.01 | yes |
| 9 | 0.825 | 0.89 | 0.1 | yes |
| 10 | 0.7625 | 0.8 | 0.04 | yes |
| 11 | 0.725 | 0.68 | 0.15 | yes |
| 12 | 0.8 | 0.83 | 0.07 | yes |
| 13 | 0.8125 | 0.97 | 0.16 | yes |
| 14 | 0.825 | 0.86 | 0.07 | yes |
| 15 | 0.8125 | 0.79 | 0.07 | yes |
| 16 | 0.8625 | 0.76 | 0.04 | yes |
| 17 | 0.775 | 0.73 | 0.01 | yes |
| 18 | 0.7625 | 0.86 | 0.07 | yes |
| 20 | 0.7375 | 0.65 | 0.16 | yes |
| 21 | 0.95 | 0.76 | 0.09 | yes |
| 22 | 0.7375 | 0.87 | 0.1 | yes |
| 23 | 0.7625 | 0.74 | 0.1 | yes |
| 25 | 0.8125 | 0.88 | 0.18 | yes |
| 26 | 0.8875 | 0.78 | 0.09 | yes |
| 28 | 0.85 | 0.74 | 0.09 | yes |
| 29 | 0.9125 | 0.71 | 0.02 | yes |
| 1 | 0.25 | 0.44 | 0.06 | no |
| 2 | 0.525 | 0.5 | 0.18 | no |
| 19 | 0.4125 | 0.48 | 0.21 | no |
| 24 | 0.325 | 0.51 | 0.09 | no |
| 27 | 0.6625 | 0.76 | 0.35 | no |
| 30 | 0.4125 | 0.67 | 0.24 | no |
|  |  |  |  |  |


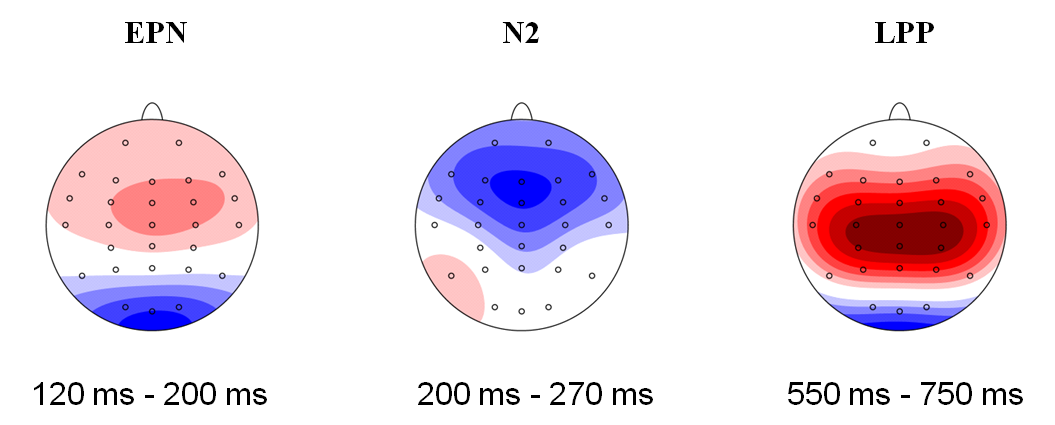


Supplementary Figure. Grand-mean ERP topographies at different time windows.
